# Apophatic science: how computational modeling can explain consciousness

**DOI:** 10.1093/nc/niab010

**Published:** 2021-06-16

**Authors:** Will Bridewell, Alistair M C Isaac

**Affiliations:** 1 Navy Center for Applied Research in Artificial Intelligence, U.S. Naval Research Laboratory, 4555 Overlook Ave SW, Washington, DC 20375, USA; 2 University of Edinburgh, Edinburgh EH8 9AD, UK

**Keywords:** computationalism, consciousness, evidence, functionalism, methodology, modeling

## Abstract

This study introduces a novel methodology for consciousness science. Consciousness as we understand it pretheoretically is inherently subjective, yet the data available to science are irreducibly intersubjective. This poses a unique challenge for attempts to investigate consciousness empirically. We meet this challenge by combining two insights. First, we emphasize the role that computational models play in integrating results relevant to consciousness from across the cognitive sciences. This move echoes Alan Newell’s call that the language and concepts of computer science serve as a *lingua franca* for integrative cognitive science. Second, our central contribution is a new method for validating computational models that treats them as providing *negative data* on consciousness: data about what consciousness is not. This method is designed to support a quantitative science of consciousness while avoiding metaphysical commitments. We discuss how this methodology applies to current and future research and address questions that others have raised.

## Introduction

“Consciousness science” is an oxymoron. On the one hand, a defining feature of consciousness is its subjective character, which we each access directly through personal experience; on the other hand, the empirical sciences demand that data be intersubjectively accessible and reproducible, effectively factoring out the personal and subjective. Paradoxically, then, the feature that distinguishes consciousness as a natural phenomenon must be absent from the data of consciousness science (Nagel 1986; Chalmers 2010; Overgaard 2015). Despite the recent explosion of empirical and theoretical work on consciousness, this paradox has yet to be adequately resolved.^1^ This paper proposes a new method for validating models in consciousness science that solves this problem. After motivating the method and its key commitments, we re-evaluate existing research in terms of our methodology and respond to a number of objections.

### Apophatic science in brief

The key insight we aim to defend is that models need not investigate an explanatory target “from within,” by exemplifying or instantiating that target. They may also circumscribe a target of explanation “from without,” by ruling out putative explanations of the target. By generating increasingly sophisticated models of cognitive agents that lack consciousness, we learn more about what consciousness *is not*, and consequently, more about consciousness itself. While this claim may appear counterintuitive, we argue that the structure of this research program is directly analogous to that found in paradigmatic quantitative sciences, such as fundamental physics. The true test of an explanatory and scientific research program is that it leads to increasingly precise models and passes increasingly rigorous evidential tests. The method we suggest conforms to this pattern and thus may ground a rigorous and quantitative science of consciousness.

Cheekily, we have christened this method *apophatic*, in reference to apophatic or negative theology (Putnam 1997). Faced with the task of understanding a divine entity, in principle inaccessible to human comprehension, apophatic theology proceeds by elucidating those features which God is not, thereby carving out a negative characterization. Likewise, consciousness exhibits essential features that are intersubjectively inaccessible, and apophatic science proceeds by systematically exploring models that fail to capture these features, thereby refuting through successful simulation insufficient accounts of consciousness. The analogy breaks down in several ways, however. Whereas God may be entirely inaccessible, consciousness is partially accessible through a wide variety of experimental paradigms. Whereas apophatic theology is used to produce subjective mystical states, apophatic science aims to produce intersubjectively verifiable empirical knowledge. Crucially, we demand that a negative characterization of consciousness be evaluable by quantitative measures such as its fit to empirical data.

### Modeling front and center

Apophatic consciousness science gives models pride of place. Inquiry is structured as follows: (i) procedures such as functional magnetic resonance imaging (fMRI), electroencephalography (EEG), and the recording of verbal report and behavioral response are used to collect data on phenomena related to consciousness; (ii) these data are summarized in mechanistic descriptions, theories, or models validated through ordinary techniques; and then (iii) these piecemeal models are integrated within a computational simulation or robotic embodiment that instantiates the relevant mechanisms and reproduces target behaviors. On our view, only step (iii) addresses consciousness *tout court*, and the new method for model validation applies only here, not elsewhere in the cognitive sciences. The challenge for consciousness science is to find a model genuinely targeted on subjective experience that can nevertheless be improved in response to intersubjective data. Apophatic model validation is a new way to assess a model’s relevance to consciousness and to iteratively improve the model through comparisons with data.

### Methodology not metaphysics

We stress that this project concerns the *methodology* of consciousness science: what are the right *methods* for studying consciousness *empirically*, and how may these methods be *justified*? Although our discussion will necessarily intersect with issues in the philosophy of consciousness, this is not a project in epistemology or metaphysics. For instance, we are not advocating *mysterianism*, the view that the relationship between consciousness and matter is somehow forever inaccessible to human understanding (McGinn 1989). That claim concerns understanding in principle and across all modes of inquiry. In contrast, we are concerned with the technical roles of intersubjective data and computational models in studying subjective phenomena. Likewise, we take no stance on whether conscious experience reduces to functional or physical properties of its neural substrate.

Although we remain agnostic on these questions, we must engage them in a limited way, as the metaphysical commitments of consciousness scientists have influenced their methodology. For instance, endorsing eliminativism or reductionism (often and implicitly) permits one to deny the subjective character of experience and to assert that models of “access consciousness” make direct progress on consciousness proper (Block 1995). Conversely, metaphysical and epistemological arguments by philosophers have been used to cast doubt on the explanatory relevance of consciousness science to phenomenal consciousness, identifying a uniquely “hard” why-question about consciousness intractable to empirical methods (Chalmers 1996). Our goal is a method that can make genuine progress on the *scientific* understanding of consciousness without taking a side in this debate.

## Computationalism and Explanatory Unity

This methodological project is grounded in two constraints on a satisfactory account of consciousness. First, we prioritize explanatory unity. Consciousness permeates human experience, and accounts that separately explain listening-to-beeps-consciousness, looking-at-squares-consciousness, reading-instructions-consciousness, Stroop-task-consciousness, car-driving-consciousness, and so on are inadequate to capture this pervasive nature. Although we may learn from such isolated explanations, the goal should always be an integrated theory that covers all *consciousness-relevant phenomena*: those behaviors, mechanisms, and scenarios identified as involving consciousness by disciplines across the cognitive sciences. Second, we embrace the methods of computational model building, which we take (without argument) to be uniquely suited to this task. More specifically, our own research aims to develop artificial, intelligent, human-like agents, and we conjecture that at a minimum, agency requires some feature to play the functional role of a conscious and unconscious divide (Bello and Bridewell 2017). These two constraints are drawn from Allen Newell’s original program for computationalism: the simulation of increasingly realistic behavior by empirically informed models (Newell 1973).

Newell initially proposed the methods of computationalism as a framework for integrating diverse types of evidence from across the cognitive sciences into coherent explanations. Consider his (1973) comments on the state of psychology in the paper “You Can’t Play 20 Questions with Nature and Win,” where he laments, “We never seem in the experimental literature to put the results of all the experiments together,” concluding that, when studying the human subject, “the ‘normal’ means of science may not suffice” (298–9). Newell was driven by the apparent heterogeneity and lack of unification across the laundry list of empirical results presented at a prominent symposium on cognition. While he found each result intriguing, he questioned whether the mere amalgamation of “effects” constitutes a genuine increase in knowledge.

Newell’s positive suggestion was that the information processing perspective could serve as the critical “glue” for binding diverse empirical results in cognitive psychology together within a unified framework. Researchers were to build complete, computational models of complex tasks or to develop wholesale “cognitive architectures” that could take instructions as input and execute a task in the same manner as human subjects. Newell does not speak specifically of consciousness, but he is interested in unifying results in cognitive psychology under a single theory that will capture “the essential structure of the mind” (ibid., 306).^2^ Accordingly, computer science was to play a critical role in Newell’s vision by providing a *lingua franca* for stating comprehensive theories of the control structures underlying human behavior. While his call has had limited traction in the cognitive sciences,^3^ we think that this *methodological computationalism* is a promising approach to developing progressively expansive and integrated models that provide a unified account of conscious experience.

## The Challenge of Modeling Consciousness

Applied to consciousness, methodological computationalism aims to construct integrated models of consciousness-relevant phenomena and to iteratively improve them in response to empirical data. As in any modeling effort, this project requires a way to validate a model (i.e., to ensure it is targeted at consciousness proper) and a way to guide its systematic improvement. These requirements are connected in that model improvements are often inspired by a failure to satisfactorily account for data. Instances of this standard modeling procedure can be found in discussions of physics (Hesse 1962; Smith 2014), biology and the cognitive sciences (Bechtel 2008; Bechtel and Abrahamsen 2010), and other fields (Morgan 2012; Currie 2018; Weisberg 2018). The unique empirical challenge of consciousness science is that consciousness is constitutively subjective, yet empirical data must be intersubjectively accessible (Chalmers 2010, Chapter 2; Overgaard 2015). We claim that there is a way to overcome this challenge in order to pursue a research program centered on computational models of consciousness. Before describing our solution, we look at this concern in closer detail.

### Confronting the hill of subjectivity

Skeptical arguments on the role of intersubjective data in consciousness science are common, so we only briefly rehearse the main points here. The qualitative discontinuity between subjective phenomenal character and intersubjective data is perhaps most clear in the case of neurological data. We agree that models may be validated against data on the neural correlates of consciousness by checking for functional correspondence between their components and corresponding neural targets. For instance, Anderson (2005) compares a computational model of algebraic problem solving with neuroscientific evidence from subjects engaged in algebraic reasoning. Yet the fit between Anderson’s model and neural data cannot validate the model *as conscious.*^4^ The most that fitting such data can do is validate a model as implementing or predicting *correlates of consciousness*, and this is precisely because we lack a theory of the causal relationship between neural correlates and consciousness itself (Chalmers 1995).

This argument extends to every method of gathering data about consciousness. Consider results on “reconstructing visual experience” from neural data (Haynes 2009); for instance, Nishimoto *et al.* (2011) report on the ability to generate images from fMRI data of a person watching a video. Their model is validated by checking the fit between the images it generates and the stimulus image, a procedure that explicitly detours around the subjective character of experience. Recently popular “no-report” paradigms (Tsuchiya *et al.* 2015) fare no better, testing for patterns of neural activity presumed to be correlated with consciousness, but confirmed only through functional analysis (Phillips 2018). Finally, in the case of verbal reports, we agree that they provide data relevant to consciousness (Dennett 2003), but as Chalmers (2018) emphasizes, they remain at best a superficial expression of the real explanandum, the consciousness-driven *disposition* to report. That is, people may be disposed to make a large number of verbal reports even though they eventually make only one, which may be specific to a current prompt or goal. As a result, the relationship between the report and the richness of the underlying conscious state is too tenuous for empirical validation.

Our purpose in rehearsing these well-known skeptical challenges for an empirical science of consciousness is not to defend pessimism, but merely to make vivid the obstacle to traditional methods of model validation. If we are dissatisfied with targeting only the correlates of consciousness, what recourse do we then have*?*

### Imagining Sisyphus happy

We see two paths to bridging the gap between models of consciousness and correlational data. On the first path, one facilitates model validation by adopting a strong metaphysical view toward consciousness, but this comes with (we think unacceptable) explanatory costs. On the second, rockier path, one embraces metaphysical agnosticism, but then must grapple with the question of how models may be validated without the possibility of confirmatory data. This move, we claim, is the more promising one although it requires the introduction of a novel methodology.

If a modeler directly identifies consciousness with intersubjectively available phenomena they can validate their model as conscious. However, by stipulating that “consciousness” is intersubjectively accessible after all, they thereby sever any evidential link between empirical data and our pre-theoretical understanding of the explanandum of consciousness science. This is an in-principle point, applying not only to functionalism,^5^ historically the preferred account of consciousness for computationalists, but also to biological materialism (Searle 1992) and panpsychism (Nagel 1979; Seager 2019). Validating a model by committing to any of these views renders model evaluation a Pyrrhic gesture, as it blocks the possibility of iterative progress toward an explanation of subjective experience. Instead, progress is made toward explaining whatever stand-in for that experience is stipulated by the theoretical view. The situation is even worse for metaphysical positions that assert that consciousness is irreducibly mysterious (McGinn 1989) or that it inhabits an empirically inaccessible realm, such as the dualism of Descartes, since they relinquish any possibility for model validation.

In contrast, agnosticism toward the metaphysics of consciousness at least avoids immediately rendering validation Pyrrhic or impossible, but a methodology for metaphysical agnosticism is far from obvious. Naïve agnosticism about whether any model itself instantiates consciousness is a nonstarter. Suppose someone builds a robot whose software architecture implements the Global Workspace Theory of consciousness (GWT; Baars 1997). True-believer GWT-functionalists assert that the robot is conscious, biological materialists and dualists assert it is not, and agnostics simply shrug their shoulders and refuse judgment. The naïve agnostic response is worse than unhelpful because it closes off any route for iterative model improvement. If no features of a model are deemed relevant to its status as conscious—as indeed must be the case for the true agnostic—then how can there be a systematic procedure for revising a model in response to evidence? If an agnostic wants to assess one model of consciousness as better than another, then they must be prepared to treat, at least provisionally, some features of models or their behavior as responsive to intersubjectively available empirical data in a consciousness-relevant way. The metaphysical agnostic needs a methodological principle for improving models of conscious agents that turns agnosticism into an evidential tool, permitting a monotonic progression of model improvement.

Consciousness science needs a *metaphysically agnostic*, but *methodologically substantive* perspective: an approach that can validate models as targeted at consciousness without relying foundationally on any position in the metaphysical debate about consciousness, thereby serving physicalists, dualists, and panpsychists equally. To be successful, this approach must ensure not only that a model is targeted at consciousness as an explanandum but also that it is susceptible to iterative improvement through comparison with empirical results. Apophatic model validation fulfills both criteria.

## The Apophatic Method

Typically, models are expected to produce output that corresponds to data measured in the world. If there is a hidden entity or process, *x*, driving the production of those data, we refer to them as *x*-relevant. Crucially, models of *x*-relevant data need not represent or reproduce *x* itself. For instance, computational models of the climate do not produce a miniature climate, they produce output that corresponds to climate-relevant data. Likewise, computational models that target consciousness as their explanandum do not need to produce consciousness to generate consciousness-relevant output.

Moving from the observation that successful models *need not* reproduce consciousness, to the provisional stipulation that they *cannot*, motivates a new methodology for model validation that supports iterative improvement: *apophatic model validation*. In this paradigm, models act as *negative data*: evidence that the theoretical principles encoded in a model of consciousness-relevant phenomena are insufficient for explaining consciousness. A model may nevertheless be assessed as an improvement over its predecessor if it reproduces more consciousness-relevant data. A sequence of “insufficient” models that each improves on their predecessors delimits the boundaries of consciousness from without, systematically probing its power to resist computational simulation.

### A science of consciousness for the metaphysically agnostic

The apophatic method combines Newell’s methodological computationalism with apophatic model validation. The core approach is in line with Newell: build a series of models that accounts for an increasing number of consciousness-relevant phenomena with increasing levels of fidelity to the mechanisms suspected of enabling human consciousness. The apophatic twist is that each model is validated according to the methodological assumption that consciousness does *not* result from information processing. In other words, a model is always inadequate, and the mechanisms that it implements are deemed insufficient for explaining consciousness. Researchers chip away at theoretical claims about consciousness-relevant phenomena through computational implementation, reporting “still not conscious” as their models increase in detail. The result is a research program that continually reports “not this, not that” about the underlying claims, challenging theorists to commit to increasingly specific hypotheses and pushing them away from dogmatic or vague assertions.

We offer apophatic model validation as a methodology that remains agnostic on the metaphysical questions of consciousness. It grants the long-standing philosophical criticisms of functionalism and its physicalist cousins (Descartes 1633; Leibniz 1714; Searle 1980; Chalmers 1996) that functionalism closes the gap between subjective experience and intersubjective data through a stipulation that renders models inadequate for targeting consciousness as we understand it pre-theoretically. Yet we do not accept the defeatist conclusion that computational methods are irrelevant for studying consciousness proper. Nor is the negative evaluation of models as “still not conscious” grounded in an anti-functionalist metaphysics. We do not assume dualism, for instance, or biological materialism, either of which would imply the conclusion that no model is conscious, at the cost of making progress in modeling consciousness impossible.

Rather, the methodological claim that no particular model instantiates consciousness should be understood *instrumentally*, as a tool for rendering data relevant to models, and thereby introducing the possibility of their progressive improvement. The irony of our ecumenical approach is this: by embracing the criticisms of computationalism leveled by anti-functionalists, we can better ground the methodological computationalism of Newell, paving the way for a consciousness science both metaphysics-neutral and responsive to data.

### An integrative science of consciousness

Notably, we do not intend to suggest that neuroscience, psychology, or any other subfield of cognitive science needs to reform either its distinctive methods or its basic strategies for model validation. Specific models in these disciplines may continue to be validated by confirming their predictions against empirical data. These techniques are unquestionably appropriate when the explanatory targets are intersubjectively measurable quantities like patterns of neural firing or behavioral profiles. However, when it comes to consciousness considered as a subjective experience, then the standards of model evaluation change. This is where apophatic models provide value, as a means both for integrating diverse results concerning consciousness-relevant phenomena and of testing claims about the nature of that integration.

By eschewing any substantive position in the metaphysical debate, the apophatic method encourages modesty. We treat the success of any integrative model of consciousness-relevant phenomena skeptically, as a negative datum that the particular pattern of functional relations implemented in the model is insufficient to account for consciousness *tout court*. This failure then motivates a successor model that subsumes an increasing amount of consciousness-relevant phenomena. Methodologically, this process proceeds by testing conjectures about what constitutes consciousness via implementation, knowing that success in modeling, which is by no means guaranteed, implies a failure to completely capture the underlying phenomenon: a “refutation by implementation.”^6^

## Apophaticism in Practice

If the apophatic approach rejects the claim that computational models may exhibit consciousness, in what sense does the project of building such models produce knowledge about consciousness? More generally, is apophatic model validation genuinely scientific? We claim that apophatic science is structurally analogous to paradigmatic instances of scientific reasoning found in physics. After elaborating this conceptual point, we illustrate our view with some concrete examples of ongoing modeling projects in cognitive science that may be recast or reformed as fragments of an apophatic science of consciousness.

### Apophatic consciousness science as a research program

We offer apophatic consciousness science as a “research program,” in the sense found in the history and philosophy of science (e.g., Laudan 1977; Lakatos 1978; Chang 2012; Smith 2014): a systematic and progressive project aimed at probing the limits of theory by constructing ever more refined models. Considered as a research program, apophatic consciousness science looks much more like paradigmatic instances of scientific inquiry, such as Newtonian astronomy or contemporary high-energy physics, than the traditional, reductive investigation of consciousness. As with any high-precision research project in the history of science, apophatic consciousness science continuously challenges the evidential support for its core theoretical commitment, in this case, the power of information processing to produce consciousness.

Likewise, just as the project of refining the evidence for Newtonian physics was coextensive with the project of finding high-quality evidence for its successor (Smith 2014), the project of building ever more refined computational models is constructive both for supporting computationalism and for supporting, even *discovering*, any successor theory of consciousness that may emerge in its wake. So long as computationalism provides the only *lingua franca* for unifying evidence from across the cognitive sciences into cognitive models, it is scientifically irrelevant whether we take its explanation of consciousness to be correct or not.^7^ As with Newtonian science in the 60-year interval between the discovery of the precession in the perihelion of Mercury and Einstein’s general relativity, we have no other choice than to continue to probe the limits of the only integrated empirical theory we have.

Of course, there is a glaring difference with the Newtonian case. We explicitly advocate a skeptical attitude toward the empirical adequacy of the computational account of consciousness, whereas nineteenth century astronomers held out hope that discovery of a new planet would explain Mercury’s apparent anomaly in Newtonian terms. Yet our choice here is purely pragmatic: as a matter of fact, computationalist theories of consciousness have proved themselves lax in probing their own limits, and all too willing to accept some particular robot or model as conscious (see below). What Newtonian science and contemporary particle physics exemplify is a strict self-discipline, a project to demand ever-increasing precision in one’s models *without end*, that we think computational cognitive science would do well to emulate.

### Existing research reassessed apophatically

Although some researchers have claimed that their computational models are conscious, apophatic methodology reinterprets this work as identifying what consciousness is not. In particular, we claim that the ability to encode the core principles of a theory of consciousness within a computational model provides evidence that the theory is incomplete. The apophatic project does not interpret successful modeling of some consciousness-relevant phenomenon as a *failure*, but rather as an invitation for theorists to refine their views and for modelers to encode this refinement in future models. We illustrate the implications of the apophatic perspective by reinterpreting the contribution of some successful computational research programs.

#### Self-consciousness and the mirror test

An agent passes the mirror test if it attempts to investigate some mark placed in secret on a non-visible part of its body (e.g., a spot on its forehead) once it views itself in a mirror. Passing the mirror test has been taken as a behavioral signature of self-awareness, or even self-consciousness. Yet, Bringsjord et al. (2015) demonstrate that mirror test passing behavior can be implemented in artificial agents. Once we recognize that the agent passes the putative test through mechanical theorem-proving, the test’s relevance to “self-consciousness” seems to evaporate. Similar considerations apply to other robotic successes at passing this test, whether they employ simple circuits (Haikonen 2007) or neural nets (Takeno 2008; Torigoe *et al.* 2009).

In a proto-apophatic manner, Bringsjord, Haikonen, and Takeno all acknowledge their implementations reveal that passing the mirror test is too trivial to serve as a measure of consciousness, taking their robots as a starting point for further research. Yet, while Bringsjord is skeptical about consciousness in artificial agents, Haikonen and Takeno argue that the robots will achieve consciousness properly at a future stage of their projects. Hastily, Torigoe et al. (2009) already declare victory in this regard writing, “We have developed a robot that is capable of consciousness and emotions similar to humans,” because it implements a custom theory of consciousness (131). From an apophatic perspective, we applaud the progressive nature of these research programs, and take each step to constitute a kind of victory; not in producing consciousness, but in demonstrating an implemented theory of consciousness as inadequate.

#### Global Neuronal Workspace Theory and the Stroop task

Next, consider Stanislas Dehaene’s extension of the Global Workspace Theory of consciousness (Baars 1997) in his Global Neuronal Workspace Theory (GNWT) and its associated computational models. A GNWT model of the Stroop task (Dehaene *et al.* 1998) takes as input two sets of four nodes: one set standing for four possible color words and the other standing for four possible colors. The neural network is trained first to distinguish inputs from either set independently, then tasked to distinguish those from the “word” inputs when interference from the “color” inputs is sent, eventually learning the task after a period of error and “effort” closely mirroring the pattern seen in human subjects (Dehaene *et al.* 1998). Reflecting on this research, Dehaene reasons, like Torigoe, that since his model implements GNWT, it is conscious: “We call ‘conscious’ whichever representation, at a given time, wins the competition for access to this mental arena (i.e., the workspace) and gets selected for global sharing and decision-making” (Dehaene *et al.* 2017).

From the apophatic perspective, we must be more specific to discover what this model reveals about consciousness. On close inspection, the workspace of Dehaene’s model is a forum where vigilance and reward signals can be integrated with input signals through reinforcement learning rather than explicit rules. The model’s success illustrates on the one hand that the Stroop task can be learned without relying on explicit rule encodings (cf. Lovett 2005), and on the other hand that conscious awareness is not necessary for Stroop-like behavior. We applaud and encourage the project to develop GNWT models that encompass wider arrays of conscious-relevant phenomena, but the apophatic perspective views the claim that any particular model in this trajectory successfully implements consciousness proper as a hinderance rather than aid to pushing this project toward broader empirical scope and increased predictive precision.

#### Architectures for consciousness

The direct descendents of Newell’s call for integrated modeling are a series of attempts to systematically account for large segments of human behavior within unified “cognitive architectures” organized around a few core principles (Kotseruba and Tsotsos 2020). Long-running projects in this tradition, such as ACT-R (Anderson *et al.* 2004) and Soar (Laird 2012) initially proceeded independently, but have lately coalesced around a Common Model of Cognition (Laird *et al.* 2017), which both encompasses shared assumptions and identifies a systematic program for expanding the capacities of a cognitive architecture. These projects satisfy the requirements we lay out for systematic, progressive model building; however, they are not instances of the apophatic method insofar as decisions about which patterns of behavioral data to reproduce next are not driven in the first instance by the question of whether that behavior is consciousness-relevant. Our own research with a computational system called ARCADIA (Bridewell and Bello 2016a), which falls loosely in this tradition, more closely conforms to the apophatic method, as our decisions about how to expand the model are targeted at a sequence of phenomena (attention, inattentional blindness, intention, and awareness) deemed relevant for approaching a progressively richer sense of consciousness (Bridewell and Bello 2016a, b; Bridewell *et al.* 2018; Bello and Bridewell 2020).

Can an apophatic science of consciousness, realized in projects such as these, genuinely explain consciousness? We do not claim it will produce explanations of how phenomenal experience *feels*, or *why* it exists. But these explanatory demands do not have analogs in any quantitative science. If instead, we seek explanations like those we find in paradigmatic disciplines such as astronomy and particle physics, then the answer is *yes*. Quantitative physics does not answer nebulous, unqualified why questions; rather, it produces a subtle and precise knowledge, of the limits of certain explanatory hypotheses, of the extent to which the world fits our best attempts at modeling it. Apophatic consciousness science is no different. By following a trajectory of increasing fidelity to neural mechanisms, simulating an increasing breadth of behavioral data, we carve out an ever more ambitious and specific understanding of what consciousness is not.

## Objections and Replies

In discussing the apophatic method with our colleagues, several comments and objections were echoed by multiple voices. To clarify our proposal and to address concerns that readers may have, we list four typical questions that we have received and include our replies.

### Is the claim: solve the easy problems first, and that will solve the hard problem?

Canonically the “easy” problems of consciousness concern consciousness-relevant phenomena, such as the reportability of mental states or the control of behavior, that may be investigated intersubjectively and explained mechanistically. The “hard” problem is the problem of explaining how (or why) physical processes give rise to phenomenal experience at all (Chalmers 1996).

Throughout our positive presentation of apophatic model validation, we have deliberately avoided the terminology of “easy” and “hard” problems. We are happy to accept a distinction between questions that may be answered empirically, through the methods of science, and those that cannot. The view outlined here is concerned purely with consciousness considered as a topic for scientific investigation. We do not wish to deny that there may be further, conceptual or metaphysical questions about consciousness that fall outside the purview of science. On which side of this boundary does the “hard” problem fall? It seems to us that the literature is unclear on this point, and this is why we have resisted the terminology of easy versus hard.

Insofar as the hard problem is understood as the question of how to empirically investigate subjectively accessible phenomenal experience, then we do intend apophatic science to make progress on it—not merely, nor in the first instance, by solving “easy” problems, but rather by systematically *integrating* piecemeal models of “easy” phenomena associated with consciousness into larger and more comprehensive models. Insofar as the hard problem is genuinely understood as a *why* question—why do certain physical states give rise to consciousness?—then we do not pretend to address it here, because it does not strike us as an empirical question. *Why* do massed bodies attract each other? This is not the question addressed by the theory of gravity; rather it gives us a quantitative account of the conditions under which massed bodies *do* attract each other.

### How is this different from heterophenomenology?

The claim that there is any *scientifically relevant* “hard problem” of consciousness has influentially been rejected by Daniel Dennett. Instead, he advocates studying consciousness through a procedure he calls heterophenomenology (Dennett 1991, 2003), which treats the verbal reports of subjects describing their inner experiences as intersubjectively legitimate data. Computational models of inner experience may be derived from careful analysis of these reports, and then, insofar as possible, checked against properties of physical correlates, potentially rejecting or amending the models on that basis. The key point being that verbal reports are not taken as veridical descriptions of the mechanisms of thought, but only descriptions of a person’s (imperfect) experience of those mechanisms.

For the most part, we agree with Dennett about the status of data. Like Dennett, we defend the legitimacy of intersubjectively accessible data as relevant to consciousness science and reject any push from mysterians or dualists that these data cannot be used to target consciousness at all. Moreover, we agree on the evidential importance of verbal reports, despite the fact that they are at most fragmentary and defeasible descriptions of experience and not necessarily an accurate report of mental processes.

However, we disagree with Dennett insofar as he sees no particular problem in validating models of consciousness by appealing to such data. Dennett views qualitative models of consciousness as validated in a traditional manner, through matching predictions with data: metacontrast masking and Libet’s neurosurgical experiments *confirm* his multiple-drafts model of consciousness and *disconfirm* the Cartesian theater (1991, Chapter 6). An implication is that Dennett takes successful implementation of a model that reproduces the full panoply of consciousness-relevant phenomena to exhaustively explain consciousness (ibid., Chapters 12–14), whereas for us, there remains a principled *evidential* gap between the subjective and the intersubjective. An apophatic interpretation of Dennett’s hypothetical sequence of models would take them as progressively shrinking the set of phenomena that are constitutively dependent upon consciousness.

### How do researchers stay motivated?

Some colleagues have expressed concern that our view is too pessimistic. If every model that accounts for consciousness-relevant phenomena will be treated as “not conscious,” then why bother building models at all? It seems as if under an apophatic science of consciousness the only way that a modeler can win is to not play the game. Addressing this concern requires a shift in perspective. Instead of asking whether a given model is conscious, we ask whether a particular consciousness-relevant phenomenon can be modeled at all. Or, more broadly, we wonder whether that phenomenon can be modeled in a way that is integrated into a model that also accounts for other proposed phenomena. *Success is not guaranteed*.

Moreover, success is by no means the most desirable outcome. Consider another analogy with physics: the “discovery” of the Higgs boson. The vast majority of physicists expected this final prediction of the Standard Model to be confirmed and would have been shocked if it had not. Anecdotally, however, their heartfelt hope was that the Higgs would not be found at the expected energy levels, or found, but with quite different properties than predicted (Kolbert 2012; Madrigal 2012). Why? Although the Standard Model is the most empirically confirmed theory in the history of science, there have been conceptual doubts since its very inception that it could possibly be correct as a description of fundamental reality (Weinberg 1997). Failure to find the Higgs, or for its properties to match predictions, would be evidence for the successor to the Standard Model. Without such evidence, in the form of disconfirmations or violated expectations, the search for a fundamental theory is in evidential limbo.

Cognitive science today is a more exciting field than fundamental physics, in part because of the great uncertainty at every turn. The possibility of consciousness-relevant phenomena that cannot be addressed through the methods of computationalism is ever present. On the one hand, this possibility challenges modelers to simulate increasingly sophisticated portfolios of conscious phenomena. On the other hand, experimentalists may be driven to seek a class of phenomena that can be specified well enough to be a legitimate target for modeling but that resists sustained efforts to encode and reproduce it. Such a discovery would identify the limits of computationalism and could point toward its most promising successor.

This observation also answers a frequently asked corollary to the question of motivation: when does an apophatic scientist know that they are done? We noticed a strong concern that researchers might one day build a model of consciousness, and the apophatic method would prevent its recognition. We suggest that there are two indicators that the research program is exhausted. First, experimentalists may no longer be able to identify new consciousness-relevant phenomena, in which case the methodological constraints of apophaticism will need to be reconsidered, and perhaps abandoned. Second, computational modelers may be challenged by a phenomenon that they cannot satisfactorily capture. In that case, we may have identified a feature of consciousness (e.g., a function, a behavioral pattern) that enables us to reject computationalism as a viable project and can serve as an evidential benchmark for its successor.

### What if the model really is conscious?

One worry that has been raised about our response regarding motivation is that apophatic science may overshoot its target: what if an early model, while failing to reproduce known consciousness-relevant phenomena, is nevertheless in fact conscious? It is worth reaffirming here that the apophatic precept is a methodological stipulation. Asserting that all successful computational implementations are not themselves conscious has value insofar as it establishes a clear route for iterative model improvement. It is not meant as a factual test, and indeed to take it as such would be to take on some of the very metaphysical assumptions we hope to avoid.

Suppose a computational model achieves consciousness. In order to be evidentially useful for a science of consciousness, there must be some method for (a) validating the model as conscious and (b) iteratively generating more data (through testing the model, or building successors similar in relevant respects) in order to improve quantitative predictions. We have argued above that traditional methods of model evaluation are inadequate to this task. So, the example is only relevant to the science of consciousness if an alternative to apophatic model validation that satisfies (a) and (b) is on offer. We would welcome such an alternative.

In our view, the real concern behind the possibility of an unrecognized conscious model is *ethical*. Destructively manipulating, erasing, or storing in isolation from any stimuli—the kinds of actions one might perform on an obsolete model—would be construed as malicious or harmful if performed on a conscious agent. We take this ethical consideration to be completely orthogonal to methodological and empirical considerations. We do not reject its importance, but rather assume that it will be dealt with by the broader social context in which the scientific community is embedded. Here, apophatic science is no different from any other empirical project. If widespread attitudes toward the ethical standing of artificial agents change, then the permissibility of existing modes of research may change, as it has in the case of animal experimentation.

## Conclusion: Everything Old Is New Again

Consciousness is a phenomenon characterized by features (subjective perspective and phenomenal character) that appear intrinsically immune to the intersubjective methods of science. Two responses to this conundrum have dominated previous debates. On the one hand, *denial*: reductive functionalism simply ignores these characteristic features, and baldly asserts that intersubjectively available patterns in information processing may be identified with consciousness. On the other hand, *mysterianism*: critics of reductionism have embraced an unsolvable “hard problem” that falls beyond the bounds of science and may only be addressed through metaphysical speculation.

The apophatic approach suggests a path forward: take the intersubjective methods of computational cognitive science to provide negative data on consciousness. We need not posit a reductive theory of consciousness to make progress on what consciousness is not. Rather, by constructing models that simulate consciousness-relevant behavior by implementing consciousness-relevant mechanisms, we can show in increasing detail the space of what can be accomplished without consciousness and thereby delineate the boundaries of consciousness as a natural phenomenon from without. In this manner, the apophatic scientist systematically chips away at the cognitive, intentional, and functional roles assigned to consciousness until all that remains is either an empty set or a circumscribed core that cannot be reduced to computational implementation. The former case would empirically vindicate methodological computationalism, and arguably change our intuitions about its reductive cousin, functionalism, while the latter would constitute a “crisis” in consciousness science (Kuhn 1970).

The apophatic method is radical insofar as it rejects the implicit functionalism that has dominated the scientific study of consciousness. Yet it is conservative insofar as it endorses the methodological computationalism that rests at the very origins of interdisciplinary cognitive science. Consequently, we suspect that much previous work on consciousness can be imported wholesale into the apophatic project. It would be a mistake to think there are no implications from the apophatic perspective for how consciousness science should proceed, however. One implication is that the project to find maximally specific neural correlates of consciousness does not seem particularly important, because isolated information processing mechanisms cannot do much explanatory work in apophatic science, which aims for richly integrated models. Likewise, there are implications for the practice of computational modeling itself. Isolated, function-specific models cannot say anything about consciousness on this perspective. Rather, only models that fall within a trajectory of increasing precision and richness as part of an expanding research program progress toward an understanding of consciousness.

We wish consciousness science to take itself seriously as a science, and this means holding itself to progressively higher standards of precision and detail when evaluating the fit between model and data. Reductive accounts fail this criterion, as they assert a link between consciousness and its correlates that cannot be validated empirically, and thus cannot be iteratively challenged and improved. In contrast, methodological computationalism, validated apophatically, is a project susceptible to iterative model improvement, and increase in precision and detail. The apophatic turn renders the science of consciousness scientific at last.

## Footnotes

1 Signs of this explosion include the proliferation of high-profile survey articles (Koch 2018; Sohn 2019), special issues such as Fazekas and Overgaard (2018) or Chella *et al.* (2019), and new journals, like *Neuroscience of Consciousness* and *Psychology of Consciousness: Theory, Research, and Practice.*

2 The idea that understanding consciousness may require modeling the system as a whole, beyond any isolated, high-level structural component, has echoes in Dennett’s (1978) call to model “the whole iguana,” and in recent challenges to coarse-grained functionalism that suggest low-level features may be just as relevant to consciousness as high-level ones (Godfrey-Smith 2016; Chirimuuta 2018).

3 We are not the only ones who advocate a return to Newell’s project; van Rooij and Baggio (2021) defend a return to Newell’s methodological computationalism, and especially the emphasis on unification and the power of computational models to generate quantitative predictions, as an appropriate response to the replication crisis in psychology.

4 Anderson (2007) claims that his cognitive architecture, ACT-R, is conscious, on the grounds that implementing a version of Global Workspace Theory makes something conscious. To his credit, he appears dissatisfied with this qualified assertion when he writes, “It still is not clear to me how invoking the concept of consciousness adds to the understanding of the human mind” (Anderson 2007, 246).

5 There is a wide variety of different flavors of functionalism (Levin 2018), and strictly speaking it may be adopted as a theory of meaning for psychological terms without pairing that with a reductionist ontology. Here, we gloss over those subtleties in order to focus on the coarse implications of this whole family of views for consciousness science.

6 We thank Ljupčo Todorovski for coining this phrase (personal communication, October 15, 2019).

7 This is a version of a claim historians have made since Kuhn. Abandoning a paradigm just because of contradictory evidence, or modeling failure, is “what scientists never do” (Kuhn 1970, 77); rather, only when a viable competing paradigm is available are they at liberty to change allegiance. Our claim is that there is at present no viable competitor to computationalism *as a lingua franca across the cognitive sciences*, and some such *lingua franca* is needed if evidence is going to be integrated from across different disciplines into unified models, as the apophatic method demands.
